# Subjectively Evaluated Effects of Domestic Violence on Well-Being in Clinical Populations

**DOI:** 10.1155/2013/347235

**Published:** 2013-02-05

**Authors:** Marika Poutiainen, Juha Holma

**Affiliations:** Department of Psychology, Psychotherapy Training and Research Center, University of Jyväskylä, P.O. Box 35, 40014 Jyväskylä, Finland

## Abstract

Effects of domestic violence are reflected in victims' physical, psychological, and sexual health as well as in victims' subjective evaluations of health or subjective well-being. The principal aim of this study was to study the extent to which the consequences of domestic violence are reflected in patients' subjectively evaluated well-being, life management, and sense of security in an emergency department, a maternity department, and a reception unit of a psychiatric hospital. A questionnaire on the effects of domestic violence was administered to 530 patients. 61 patients reported either current or previous domestic violence that affected their current well-being and life management. Domestic violence was reported to have an effect on subjective well-being and sense of security: the more recent or frequent the experience of violence was, the greater was considered its impact on well-being and sense of security. Routine inquiry can uncover hidden cases of abuse and hence would be of great benefit in the healthcare context. Early identification of abuse victims can prevent further harm caused by violence.

## 1. Introduction

Findings from previous studies suggest that violence increases the use of all health services [1, 2]. As a result, the prevalence of domestic violence is assumed to be higher in clinical settings than in the general population. A review of prevalence studies on domestic violence against women found high figures for violence in obstetrics and gynaecology, psychiatric, and emergency clinic settings [3]. Results for the same Finnish clinical populations as in this study [4] indicate moderate to high prevalence rates of both recent and lifetime domestic violence. In the maternity department, nearly 3% reported experiencing current abuse, whereas over 29% reported previous abuse. These prevalences mirror some previous findings [5], but are lower than others [6]. Among the emergency department respondents, 10% reported current domestic violence and over 20% past experience of abuse. Similar lifetime prevalences have been reported, for instance, by Boyle and Todd [7]. The highest incidence and lifetime prevalence rates were found among the patients in the psychiatric hospital: current domestic violence was reported by 29% and previous violence by 51% of these patients. These findings are also consistent with those found in other studies [8].

Although violence increases the use of all health services, studies have shown that victims of abuse often seek medical help for other health complications than abuse-related injuries [9]. Findings from previous studies suggest that female victims may delay seeking help until the abuse becomes extremely serious [10]. Two-fifths of the women killed by their intimate partner had sought medical care in the year prior to their murder [11]. Moreover, victims of abuse may be reluctant to discuss their experiences: evidence suggests that the disclosure of abuse is related to being directly asked about it [12, 13]. Such low rates of spontaneous disclosure suggest that the responsibility for the identification of domestic violence may lie with healthcare professionals. However, there is a tendency for health professionals to focus on fixing the injuries and consequences of domestic violence and bypassing violence as the cause of symptoms and injuries [14]. Routine inquiry may be of great benefit in such situations.

Routine inquiry—also referred to as universal screening—is defined as inquiry about domestic violence targeted at all patients in a given setting [15]. Not only does routine inquiry have many advantages [16], it is also accepted by the victims of abuse [17, 18]. Routine inquiry increases the detection of violence victims by uncovering hidden cases of abuse; it makes access to support services easier for victims and helps maintain the safety of the victim [16]. In addition, routine inquiry changes health professionals' knowledge and attitudes towards domestic violence. It is commonly suggested that healthcare services constitute an ideal setting for screening for domestic violence because of the frequent contact of personnel with possible violence victims.

## 2. The Consequences of Domestic Violence for Well-Being

Domestic violence has adverse effects on a victim's physical, psychological, and sexual health regardless of the type of violence experienced. Studies have shown that the consequences of domestic violence can last long after the violence has ended [19]. Injuries, stress, and fear caused by domestic violence can lead to more chronic health problems, such as chronic pain, recurring central nervous system symptoms, and differential gastrointestinal symptoms and disorders [20]. Among possible psychological conditions, Macy and colleagues [2] reported that domestic violence can result in depression, anxiety, and posttraumatic stress disorder (PTSD). Victims of abuse are also more likely to have co-occurring mental illnesses. Moreover, the previous research suggests that there is a significant relationship between child abuse and different adult psychological disorders, including psychosis [21].

Researchers suggest that the timing, frequency, and type of violence experienced affect the overall magnitude of the impact of domestic violence on the health of victims. For instance, Wijma et al. [22] concluded that the recency of violence is related to the frequency of PTSD symptoms among physically or sexually abused women. Similarly, Macy and associates [2] found that recent violence has a more detrimental effect on health compared to previous domestic violence. A study conducted by Tolman and Rosen [23], in turn, indicated that women who had experienced abuse recently (within the past 12 months) were more likely to have a mental health disorder than the past-victim group (women who have not been abused in the past 12 months) and also reported more health-related concerns than nonabused women. Thus, although the impact of violence can be enduring [19], studies have shown that its effects may diminish over time if the abuse is not repeated [23]. 

Different types of violence may also have different consequences on individual well-being. Sexual abuse in particular is considered to be detrimental for women's physical and psychological health [2]. Furthermore, the impact of victimization may be greater if the violence takes multiple forms [2, 20]. In addition, studies suggest that severe and chronic forms of violence have the most serious effect on individual well-being [24]. 

It has been suggested that men and women have different interpretations of domestic violence and its consequences. In a study conducted by R. P. Dobash and R. E. Dobash [25] the results indicated that men's violence toward women differs from women's violence toward men not only in terms of frequency, severity and, consequences, but also in terms of the victim's sense of safety and well-being. Most men in the study reported women's violence towards them to be “inconsequential,” “not so serious,” or “slightly serious” and indicated that the violence did not affect their sense of well-being and safety [25, page 343]. Differences between these interpretations may be caused by the fact that men's and women's experiences of domestic abuse differ qualitatively and quantitatively [26]. Violence against women appears to be more frequent and severe, and women are also at greater risk for sexual violence or coercion [25–27]. Accordingly, some studies have suggested that the physical and psychological consequences of domestic violence may be more severe for women than men [28]. 

## 3. Subjective Well-Being

Violence adversely affects the victim's quality of life along multiple dimensions [2, 20, 29]. The detrimental effects of violence are reflected not only in victims' physical, psychological, and sexual health, but also in victims' subjective evaluations of health or subjective well-being (SWB). Studies have shown abuse victims to report their current health as “poor” or “very poor” more often than individuals who did not report abuse [19, 24, 30].

According to Friedman et al. [31, page 189] subjective well-being (SWB) refers to “the psychological well-being of a person and how satisfying a person believes his or her life is.” In other words, SWB is a person's global assessment of quality of life, as measured from that individual's personal perspective [32, 33]. SWB is comprised of cognitive and affective components, involving one's cognitive judgment of overall life satisfaction and positive and negative emotional reactions to one's life, respectively [32, 34].

Research has shown that in addition to factors such as age, gender, income, and mental and physical health, life events also have a significant influence on SWB [33, 35]. Whereas positive events seem to increase one's SWB, negative events have a decreasing or negative impact. It is important to note that one's perceived ability to control life events is related to the overall impact an event has on one's SWB [35]. This suggests that life management or the relatively stable belief that one has the ability to control one's life and the things that happen to one will also contribute to one's SWB by moderating the effects of different life events.

Previous studies suggest that subjectively rated health has a strong relationship with happiness and SWB, whereas the relationship between objectively evaluated health and SWB is substantially weaker (yet statistically significant) [35]. It can thus be argued that an individual's subjective evaluation of well-being offers an alternative approach to more objective health measures (such as symptom checklists or physician ratings) in examining the effects of domestic violence on health and well-being. Subjective evaluation of well-being represents a global evaluation of life satisfaction, involving both physical and psychological aspects of health and the subject's affective reactions to life events. Although subjective well-being has been widely researched in different contexts [33, 35], the question of the influence of domestic violence on subjectively evaluated well-being has not received much interest.

## 4. Objectives

In most cases, the consequences of domestic violence for physical and psychological health have been studied using survey questionnaire methods. A common approach to addressing the relationship between violence and adverse health consequences is to present victims with questions about their life-time experiences of violence and recurrent health problems. Only few studies to date have also included victims' subjective opinion of their current health (e.g., [19, 24, 30]).

Wijma and colleagues [22] used subjective evaluations in order to compare violence experiences of women with PTSD and women who did not suffer from PTSD. Using a Likert- type scale ranging from 0 to 10, they asked female abuse victims to mark the number that best corresponded to their experience of abuse, when it happened, and how much they had suffered from that abuse. The results led to the conclusion that the more traumatic the experience was appraised as being, the more suffering it caused and the greater the risk for developing PTSD. PTSD, in turn, was found to be associated with the amount and recency of the abuse. Unfortunately, the possible influences of timing, type, or frequency of violence on victims' evaluations were not examined. Evidence further suggests that there are individual differences among abuse victims in their interpretation of the consequences of violence. For instance, Wijma and colleagues [6] found that not all abuse victims report ill effects experienced as a result of the experience of violence. However, the possible influences behind these differences were not thoroughly addressed in this study either.

Given the gap in the literature pointed out above, this study focuses on subjective evaluations by the victims of domestic violence on its effects on their current well-being, life management, and sense of security. In particular, the overall objective of the study was to examine the factors influencing these subjective evaluations. It was hypothesized that these subjective evaluations would be moderated by the factors that influence the magnitude of the impact that violence has on health. These factors, which have been identified earlier, are the timing, frequency, and type of violence. The potential impact of gender differences and differences in the subjective evaluations between the three patient groups are also examined.

## 5. Methods

The effects of domestic violence were measured using a routine inquiry tool in the Violence Intervention in Specialist Healthcare (VISH) project. VISH is the first comprehensive project designed specifically for the social and healthcare sector in the Finnish context. It has been funded by the EU Daphne Programme in 2009-2010 and by Central Finland Health Care District (CFHCD). Its main objective is to improve early identification of and intervention in domestic violence in the District's hospitals by increasing the skills, ability, and willingness of the staff to ask about domestic violence and, if required, to intervene. In so doing, VISH will provide an evidence-based model for intervening in domestic violence in specialist healthcare settings and strengthen the channels for offering help to both the victims and perpetrators of violence and their families. 

One of the specific research aims of the VISH project was to examine the prevalence, incidence, and nature of domestic violence in three pilot units: an emergency department (ED), a gynaecology department, and the acute unit of a psychiatric hospital. Data were collected during the VISH study, which focused on the identification of adult victims of violence, and further care assessments made by staff form the foundation for the present paper.

 In the VISH project domestic violence is defined as abuse committed by current or former spouses, boy- or girlfriends, parents, step-parents, siblings, grandparents or other close relatives of the victim. The following types of violence are included: physical, emotional, sexual, religious/cultural, and economic violence, as well as neglect of care. 

### 5.1. Measuring the Effects of Domestic Violence

The routine inquiry tool was developed on the basis of international screening instruments, such as the Abuse Assessment Screen (AAS) [36], the Hurt, Insult, Threaten and Scream (screening tool) scale (HITS) [37], the Partner Violence Screen (PVS) [38], and the Woman Abuse Screening Tool (WAST) [39]. Also considered in the design of the routine inquiry tool were the recommendations for data collection on domestic violence of The National Institute for Health and Welfare (THL) and The Council of European Member States. The documentation of domestic violence is recommended to at least include the following information: (1) age and (2) sex of the victim; (3) age and (4) sex of the perpetrator; (5) the relationship between the victim and the perpetrator; and (6) the type of violence (e.g., according to the International Classification of Diseases, ICD-10) [40].

The questionnaire includes both filter and mapping questions for healthcare professionals when interviewing patients about their domestic violence experiences. In the VISH study, questions were posed to respondents orally in a private space by members of the hospital staff, such as nurses or midwives. The filter questions addressed current or previous domestic violence experiences and their effect on the well-being and life management of the patient. Victims of abuse were asked to evaluate the effects of violence on their well-being, life management, and sense of security Respondents who reported that their well-being and life management were affected were invited to answer mapping questions, that is, more detailed questions about the violence they had experienced, and asked to estimate the extent to which this has affected both their current well-being and sense of security. The latter was assessed on an 11-point scale (0 = no effect, 10 = serious effect). The mapping questions also addressed the type of violence, perpetrator, latest time, and frequency, and whether or not children had witnessed the abuse. In addition, the healthcare professional conducting the interview was asked to evaluate whether the victim being interviewed was exposed to significant health risks in his or her current situation.

### 5.2. Setting and Subjects

The data collection was conducted during the Spring of 2010 among three VISH pilot units in the CFHCD. During set periods, patients from these pilot units were routinely questioned by means of a structured form about their experiences of domestic violence. Patients meeting the following excluding criteria were not included in the study: age under 18 years old; mother language other than Finnish or insufficient language skills in Finnish; disabled or handicapped; and resident outside the CFHCD. 

 Of the 530 patients presented with the filter questions, 61 patients indicated that current and/or previous domestic violence affected their current well-being and life management. These 61 individuals were further interviewed by means of the mapping questions. 

### 5.3. Analysis

The analysis was conducted using the Predictive Analytics Software (PASW) Statistics 18.0 Package for Windows. This involved, first, comparisons of the different patient and gender groups based on respondents' verbal evaluations of the influence of domestic violence on their current well-being and life management. These comparisons were achieved by the means of the Pearson's Chi-Square (*χ* ^2^) test and the Fisher-Irwin Exact Test (*P* < .01). The Pearson's *χ* ^2^ was used where the relevant assumptions were satisfied (i.e., for all tables in which more than 25% of the expected counts were greater than five), and in all other cases the Fisher-Irwin Exact Test was used. 

The comparison between patient groups with regard to their numerical evaluations of the influence of domestic violence on both well-being and sense of security was conducted using the Kruskal-Wallis one-way Analysis of Variance (ANOVA). The variables of interest were not normally distributed, and therefore this nonparametric test was used (as opposed to the parametric ANOVA). Pairwise comparisons were conducted using the Mann-Whitney *U* test. 

Finally, the relationship between respondents' subjective evaluations and the specified background variables was studied using cross-tabulation and correlation coefficients, namely, the Pearson's Product-Moment Correlation Coefficient and the Spearman Rank Order Correlation Coefficient. Where sample sizes and the scale of the variables precluded the use of the parametric Pearson's correlation, these were computed solely to determine whether the Spearman coefficients resembled them sufficiently to justify the use of Regression Analysis. 

Hierarchical Regression Analysis (RA) was used to enable the identification of the relative importance of each independent variable in accounting for the observed variance in the independent variables.

## 6. Results

Nearly all of the victims of current violence (21/22) reported that the most recent experience of abuse influenced their current well-being and life management. Patients in the ED were not presented with the question as during tests of the tool in the psychiatric hospital and the maternity department; it was found that current abuse was reported to have an effect on well-being and life management in nearly in all cases. However, only 38% (59/155) of the victims of previous violence (ED patients included) reported that these past experiences influenced their current well-being. The percentage difference between the impact of recent versus past experiences on well-being and life management was statistically significant (*z* = 5,08; *P* < .001). Thus, experiences of abuse were more often evaluated as impacting on the well-being and life management of victims of current violence than victims of previous violence. 

### 6.1. Subjective Evaluations of the Effects of Domestic Violence

Although within each patient group small percentage differences were observed with regard to the number of victims who evaluated previous domestic violence as influencing their well-being and life management, no statistically significant differences were found between the three patient groups (*χ* ^2^(2) = .19, *P* > 0.99).

Gender comparisons were conducted between the ED and the psychiatric hospital. In the ED, only one male victim of previous domestic violence reported current ill effects from this abuse. The corresponding rate for females was 41% (7/17). However, no statistically significant gender differences were found among the ED patients (*P* = .61, Fisher's Exact Test).

In the psychiatric hospital, in turn, 25% (3/12) of male victims and 56% (5/9) of female victims reported that previously experienced domestic violence influenced their current well-being and life management. In spite of the percentage difference, no statistically significant difference was found. Male and female patients in the psychiatric hospital did not differ in their evaluations of whether or not previously experienced violence affects their current well-being and life management (*P* = .20, Fisher's Exact Test).

During the mapping questions, victims who had stated that their experience of abuse, current or previous, had impacted on their current well-being and life management were asked to estimate on an 11-point scale (0 = no effect, 10 = serious effect) the extent to which the abuse has affected both their current well-being and sense of security. The medians of victims' subjective evaluations were 3 maternity patients, 9 psychiatric patients, and 10 ED patients.

The Kruskal-Wallis test showed significant differences between the patient groups' numerical subjective evaluations of the effects of domestic violence on well-being (*χ* ^2^(2) = 15.6, *P* < .001). Further analysis through pairwise comparisons showed significant differences between the maternity department and the ED (*U* = 61,5, *P* = .005) as well as the maternity department and the psychiatric hospital (*U* = 100,5, *P* = .001). No differences were found between the subjective evaluations of the ED and psychiatric patients (*U* = 46,5, *P* = .70). The effects of domestic violence on well-being and life management were evaluated more negatively by the ED and psychiatric patients than maternity patients. Finally, no differences were found between the patient groups in their subjective evaluations of the effect of domestic violence on sense of security (*χ* ^2^(2) = .92, *P* = .63).

### 6.2. Variables Influencing Subjective Evaluations

The subjective evaluations of the impact of domestic violence on well-being or sense of security were influenced by only one type of violence, namely, neglect of care. Only one participant did not report experiencing emotional abuse.

A significant association was found between the well-being evaluation and the security evaluation (*ρ* = .55, *P* < .01). With regard to the other background variables, the subjective evaluation of the effects of violence on well-being was found to be negatively correlated to the recency (*ρ* = −.53, *P* < .01) and frequency of violence (*ρ* = −.40, *P* < .01). This finding suggests that victims considered violence to have a higher impact on well-being if it was more recent or frequent. The subjective evaluations of the effect of violence on sense of security were also influenced by the most recent experience of violence (*ρ* = −.51, *P* < .01) and frequency of violence (*ρ* = −.29, *P* < .05).

As is apparent from Table 1, the nonparametric correlation coefficients resembled the parametric coefficients sufficiently, and thus RA was conducted. Here, a hierarchical regression model was conducted, first for the sense of security evaluation. The reasoning behind this decision was based on the strong association between the two subjective evaluations and the possibility that the sense of security evaluation (which can also indicate fear) has an impact on the well-being evaluation. Variables were entered into models based on correlations.

The most recent experience of violence served as a significant predictor of the security evaluation (*β* = −.59, *t*(59) = −5, 57, *P* < .001) and explained 34,5% of the variance in these evaluations (*F*(1,59) = 31,04, *P* < .001). When the effect of the most recent experience of abuse was accounted for, the frequency of violence did not significantly increase the proportion of the variance explained (Δ*R* ^2^ = .003, *F*(1,58) = .25, *P* = .62). Consequently, this variable was excluded from the final model. Similarly, neglect of care was not included in the model, because one of the correlation coefficients was statistically significant. 

The variables entered into the regression model for the well-being evaluation were the security assessment, most recent experience of violence, frequency of violence, and neglect of care. The analysis indicated that when frequency of violence was entered into the model as a second predictor, more variance was predicted (Δ*R* ^2^ = .06) than when the most recent experience of violence was entered (Δ*R* ^2^ = .045, ns). Thus, the final regression model included the sense of security evaluation (*β* = .44, *t*(57) = 4,25, *P* < .001), frequency of violence (*β* = −.31, *t*(57) = −3,10, *P* = .003), and neglect of care (*β* = .28, *t*(57) = 2,78, *P* = .007). This model explained a significant proportion (50%) of the variance of the well-being evaluation.

## 7. Discussion

The consequences of domestic violence were clearly reflected in victims' subjective evaluations. Moreover, nearly all the victims of current domestic violence reported the abuse to influence their current SWB and life management. Previous results have suggested that some types of domestic violence, especially sexual abuse, may have more detrimental consequences than others for the health of the victim [2]. However, in the present study, subjective evaluations of both well-being and sense of security were influenced by only one type of violence, namely, neglect of care. Thus, no significant association was found between sexual violence and the subjective evaluations. A possible explanation for this finding is that most of the victims of sexual violence reported the most recent experience of abuse to be earlier in adulthood or in their childhood, whereas over one-half of the victims of neglect of care reported experiencing violence within the past 24 hours, week, or month. As previous research has indicated, the traumatic consequences of abuse may diminish if the violence is not repeated [23]. For instance, victims of previous abuse may have already received help and treatment in their situation [6]. 

As already suggested above, the recency of violence was related to the subjective evaluations of both well-being and sense of security. Experiences of abuse were more often evaluated as impacting on their well-being and life management by victims of current domestic violence than victims of previous abuse. This finding is in accordance with findings from previous research which have suggested an association between the most recent experience of violence and its consequences for health [2, 6, 22, 23]. In addition, significant associations were found between both of the numerical subjective evaluations and the frequency of violence. This finding is also in accordance with previous research, which has indicated that the effects of violence may be more severe if the violence experienced is chronic [24]. Together these results lead to the conclusion that the more recent or frequent the violence was, the higher its impact on SWB and sense of security was perceived to be. In this regard, a significant relationship was also found between the two different subjective evaluations. A possible explanation for this finding is that the fear caused by violence influences the victim's sense of security, which in turn has an effect on overall SWB. 

Finally, when the relative importance of each associated variable (the type, timing, and frequency of violence) and the relationship between the subjective evaluations were controlled, the results indicated that the subjectively evaluated effects of domestic violence on sense of security were mostly influenced by the most recent experience of violence. This explained more than a third of the variance in the dependent variable. SWB, in turn, was influenced by the sense of security evaluation, the frequency of violence, and experiences of neglect of care. Together, these variables accounted for over 50% of the variance in SWB. 

This study also examined possible differences with regard to the subjective evaluations by gender and patient group. In the patient groups, the effects of domestic violence on SWB were evaluated more negatively by the ED and psychiatric patients than maternity patients. One possible explanation for this finding is that most of the maternity patients reported experiencing violence earlier in adulthood or childhood, whereas for most of the ED and psychiatric patients the last experience of abuse was more recent. No significant gender differences were found.

## 8. Limitations and Strengths

The number of participants answering the mapping questions was small (*n* = 61), and the comparisons between the gender and patient groups were done for even smaller subgroups. Although a significant difference was found between the patient groups in their subjective well-being evaluations, these results must be interpreted with caution. 

The data collection has some potential limitations. For instance, the answers given by the participants may have been influenced by the style in which the screening was conducted. Whereas in the present paper the questions were presented to the participants orally, some studies have suggested that victims of violence prefer self-administered questionnaires over face-to-face interviews [41]. However, this matter is controversial, since other studies have reported higher detection rates with verbal screening compared to self-administered written screening [42]. Also, since the data collected in this study rely solely on self-reports, the general unwillingness to disclose violence, possible recall bias, or suppression of traumatic memories may have influenced the findings [6, 43]. In the case of maternity patients, for example, mothers often fear that talking about domestic violence may cause them to lose custody of their children [10, 44, 45].

Despite the possible weaknesses mentioned above, this study also had a number of strengths worthy of consideration. For example, the present routine inquiry targeted at victims of domestic violence was comprehensive and aimed at reaching as many abuse victims as possible. The focus was not only on intimate partner violence (IPV, i.e., abuse committed by current or former spouses), but also on abuse committed by parents, step-parents, siblings, grandparents, or other close relatives. In addition, a wide variety of violent acts were screened for unlike several other studies, which have concentrated on the most common forms of abuse (i.e., psychological, physical, and sexual), this study also focused on victims of neglect of care, religious or cultural and economic violence. Finally, the abuse experiences of both sexes were examined, as previous studies have suggested that domestic violence affects both men and women. 

## 9. Conclusions

This study adds to the literature by indicating the presence of high incidence and lifetime prevalence rates of domestic violence among clinical populations. Most of these incidences, current or past, would have remained concealed had this study not been conducted. It may therefore be concluded that routine inquiry into abuse experiences may be of great benefit in the healthcare context. As the previous literature has suggested, routine inquiry uncovers hidden cases of violence, changes perceptions of the acceptability of domestic violence, and makes support services more accessible to victims, while at the same time assisting in maintaining their safety [16].

This study also addressed a specific gap in the previous research, namely, the relationship between violence and subjective well-being. The results of this study indicated that the consequences of abuse are strongly reflected in the subjectively evaluated well-being, life management, and sense of security of the victim. The more recent and frequent the abuse was, the higher its impact on well-being and sense of security was evaluated to be. Although intervention at any point can be regarded as effective, since the effects of the abuse can be enduring [29], the results of this study underline the importance of early detection of victims of domestic violence. Such early detection can prevent violence from escalating in severity and causing its victims further harm.

## Figures and Tables

**Table 1:** Summary of Spearman and Pearson's correlations for scores on well-being, security, recency, frequency, and neglect of care.

Measure	1	2	3	4	5
(1) Well-being	—				
(2) Security	.549**	—
	(.609**)	
(3) Recency	−.534**	−.511**	—
	(−.530**)	(−.587**)	
(4) Frequency	−.404**	−.279*	.623**	—
	(−.418**)	(−.291*)	(.569**)	
(5) Neglect of care	.380**	.244	−.314*	.068	—
	(.386**)	(.293*)	(−.356**)	(.058)	

Significance level: *P < .05, **P < .01

Pearson's correlations are presented in parenthesis.
